# AI in breast cancer screening: a critical overview of what we know

**DOI:** 10.1007/s00330-023-10530-5

**Published:** 2023-12-21

**Authors:** José Luis Raya-Povedano

**Affiliations:** 1 Maimónides Biomedical Research Institute of Córdoba (IMIBIC), Córdoba, Spain; 2 Breast Cancer Unit, Department of Diagnostic Radiology, Reina Sofia University Hospital, Menéndez Pidal Avenue s/n, 14004 Córdoba, Spain; 3 University of Córdoba, Córdoba, Spain

Population-based breast cancer screening programs with mammography have proven to be the most effective method for reducing breast cancer mortality (by up to 30–40% of participating women) and are implemented in most European countries.

Despite their undoubted benefits, they are not free of problems. The main ones are false negatives (cancers not detected in screening readings) and false positives (recalls due to benign findings). The reading of screening mammograms is a very heavy workload for examinations that, in most cases, will be normal. Double reading and tomosynthesis, used to reduce false negatives, multiply this workload. This problem is even greater in a setting where there is a lack of expert breast radiologists.

Artificial intelligence (AI) applied to breast imaging, with new algorithms based on deep learning, has undergone enormous development in recent years. These algorithms are capable not only of identifying lesions in mammography and tomosynthesis studies, but also of assigning a degree of suspicion to each finding and to the overall study. This capability allows sorting and classifying the studies according to the likelihood of cancer being present.

The main utility of these AI algorithms in breast cancer screening lies in their ability to reduce the reading workload by fully or partially replacing human reading. But to be safely applied to the screening workload, it should not lead to a decrease in cancer detection or an increase in false-positive recalls.

In the last four years, multiple retrospective simulated studies have been published studying this ability to safely reduce workload in long screening series. These studies involve different devices, different AI commercial software, and different countries. The first conclusion that can be drawn from them is that AI adequately classifies studies according to cancer risk. In a series of 122,012 mammograms and 752 cancers diagnosed in screening, Larsen et al [1] found that 86.8% of the cancers were in the 10% of studies classified by the AI as the highest risk and only 4.4% in the 70% of those with the lowest suspicion. Other publications obtained similar results. Knowing this distribution of cancers, these studies simulated different ways of applying the AI and compared the results with the original readings.

Some of these studies assess the ability to consider negative and dismiss low-risk studies for human readings. The retrospective study published by our team in 2021 [2] concluded that excluding for reading 70% of these studies considered by the AI as low risk did not result in a loss of sensitivity in the detection of cancers or an increase in false-positive recalls.

Other retrospective studies simulated various combinations in which AI was used to replace human readers fully or partially [3–5]. In general, the conclusion of these publications is that the best application of AI is in combination with human readings, either by replacing one reader [3, 4] or by including AI in a decision algorithm involving the reader [5].

Some of these studies also included interval cancers. Lang et al [6] demonstrated that most of the interval cancers considered false negative were retrospectively classified by the AI in the highest risk scores and could potentially have been detected prospectively with the help of the AI.

Evidence about the performance of AI in digital breast tomosynthesis (DBT) is less robust than in digital mammography (DM), and the results are, to date, worse. Our retrospective publication about stand-alone use of AI in DM and DBT [7] concluded that in DBT, the performance was inferior to that of the original readings. Several reasons have been mentioned for the inferiority of AI in DBT. First, there are fewer sets of tomosynthesis studies to train the algorithms. Second, technically, the analysis of tomosynthesis is more complex, and third, there are many differences in the characteristics of the images obtained by the different manufacturers, which limits the extrapolation of the results.

Although the studies published to date report better performance of AI algorithms in DM than in DBT, the conclusions of two retrospective simulated studies in paired DM-DBT screening series [2, 8] suggest that AI-assisted DBT screening, excluding low-risk studies from reading, could replace DM screening with a lower reading workload, higher cancer detection, and fewer false-positive recalls.

The main limitation of these retrospective studies is that, although they are based on unenriched series, decisions are simulated, and it is not possible to know what the reader’s behavior would have been if they had known the result of the AI when reading.

Two recently published prospective studies support the results of the retrospective studies. In the preliminary results of their randomized study (MASAI), Lang et al [9] demonstrated that detection of cancers was non-inferior and recall rate of false positives was not higher when 90% of lower suspicion studies were single read and only 10% of higher suspicion studies were double read. In the second, Dembrower et al [10] demonstrated higher sensitivity in detecting cancers when the AI was used to replace the second reader as compared to human double reading. The number of studies sent to consensus was higher, but not the final recall rate.

Prospective studies provide strong support for the incorporation of AI in breast cancer screening, with some limitations. Their good results are the combination of the automatic behavior of AI with the final decision of radiologists trained in the use of AI and aware of its strengths and weaknesses and may not be generalizable to other centers.

The application of AI in screening opens great possibilities and poses some challenges. It will facilitate the extension of DBT screening and may help to extend screening to populations where there is a lack of breast radiologists. The use of AI will also demand expert radiologists and advanced diagnostic techniques to manage recalled woman because of increasingly subtle findings. Finally, ethical and legal issues cannot be overlooked, particularly when AI is used to exclude human reading from all or part of the studies.

## Declarations

### Guarantor

The scientific guarantor of this publication is José Luis Raya-Povedano.

### Statistics and biometry

No complex statistical methods were necessary for this paper.

### Methodology

• commentary
